# The Effect of Serum *β*-Human Chorionic Gonadotropin on Pregnancy Complications and Adverse Pregnancy Outcomes: A Systematic Review and Meta-Analysis

**DOI:** 10.1155/2022/8315519

**Published:** 2022-09-09

**Authors:** Ju Huang, Yuying Liu, Hua Yang, Yuanfang Xu, Wei Lv

**Affiliations:** ^1^Department of Obstetrics, People's Hospital of Wanning, Hainan 571500, China; ^2^Department of Clinical Laboratory, Haikou Hospital of Traditional Chinese Medicine, Haikou 570216, China; ^3^Department of Clinical Laboratory, The Second Naval Hospital of Southern Theater Command of PLA, Sanya 572000, China; ^4^Department of Gynecology, People's Hospital of Wanning, Hainan 571500, China

## Abstract

**Background:**

The relationship among elevated serum *β*-human chorionic gonadotropin (*β*-hCG), the incidence of pregnancy complications, and adverse pregnancy outcomes has been controversial. Differences in study design, subject bias due to demographic characteristics, and differences in local medical levels could contribute to inconsistent results.

**Methods:**

Literature searches were performed in PubMed, EMBASE, Medline, Central, China National Knowledge Infrastructure (CNKI), Wanfang, and China Science Digital Library (CSDL) databases. Inclusion criteria were as follows: (1) research subjects were singleton pregnant women; (2) the study is identified as cohort study; (3) the subjects were assigned to the high *β*-hCG group and control group according to whether the exposure factors increased *β*-hCG in the second trimester; (4) the observed outcomes include at least pregnancy-induced hypertension (PIH), diabetes (gestational diabetes mellitus, GMD), preterm delivery (PD), and intrauterine growth restriction (IUGR); and (5) the odds ratio (OR) and 95% confidence interval (CI) of exposure factors are calculated based on literature dataset. To determine the risk bias of selected literatures, Newcastle-Ottawa scale was applied. The chi-square test was further used for heterogeneity analysis. If heterogeneity was identified, subgroup analyses were then performed for source investigation.

**Results:**

A total of 13 literatures were included and analyzed, including 67,355 pregnant women and 5980 pregnant women assigned to the high *β*-HCG group and 61,375 pregnant women to the control group. The incidence of PIH in the high *β*-HCG group was higher than that in the control group (OR = 2.11, 95% CI [1.90, 2.35], *Z* = 13.85, *P* < 0.00001). There was no heterogeneity among literatures (*χ*^2^ = 8.53, *P* = 0.38, *I*^2^ = 6%), and thus there is no identified publication bias (*P* > 0.05). The incidence of preterm birth in the high *β*-HCG group was higher than that in the control group (OR = 2.11, 95% CI [1.90, 2.35], *Z* = 13.85, *P* < 0.00001). The analysis suggested no heterogeneity among included literatures (*χ*^2^ = 11.78, *P* = 0.11, *I*^2^ = 41%) and no publication bias (*P* > 0.05). Higher incidence of abortion was observed in the high *β*-HCG group compared with the control group (OR = 2.80, 95% CI [1.92, 4.09], *Z* = 5.32, *P* < 0.00001). There was no heterogeneity among literatures (*χ*^2^ = 3.43, *P* = 0.33, *I*^2^ = 13%) and no publication bias (*P* > 0.05). The incidence of gestational diabetes was higher in the high *β*-HCG group than in the control group (OR = 2.15, 95% CI [1.05, 4.40], *Z* = 2.09, *P* = 0.04). Heterogeneity was identified among literatures (*χ*^2^ = 47.01, *P* < 0.00001, *I*^2^ = 87%). Sensitivity analysis showed that the results were not robust, and there was no publication bias (*P* > 0.05). Compared with control, the incidence of IGUR was higher in the high *β*-HCG group (OR = 2.70, 95% CI [1.75, 4.19], *Z* = 4.45, *P* < 0.0001) with no heterogeneity among literatures (*χ*^2^ = 3.92, *P* = 0.14, *I*^2^ = 49%) and no publication bias (*P* > 0.05).

**Conclusion:**

High levels of *β*-hCG during pregnancy in singleton women are associated with a high incidence of pregnancy complications and adverse pregnancy outcomes. Pregnant women with high levels of *β*-hCG should be monitored more closely, followed up, and given timely medical interventions to reduce the incidence of pregnancy complications and adverse outcomes.

## 1. Introduction

Common pregnancy complications and adverse pregnancy outcomes, including pregnancy-induced hypertension (PIH), diabetes mellitus (GMD), preterm delivery (PD), intrauterine growth restriction Intrauterine growth restriction (IUGR), and miscarriage, are important causes of increased maternal and perinatal morbidity and mortality [1–3]. In addition, pregnancy complications and adverse pregnancy outcomes increase family burdens and consumption of social medical resources [4, 5]. Due to the variety in the level of medical technology, prevention of complications during pregnancy and safe delivery remains challenging especially in developing countries. Accurate prediction and comprehensive monitoring and follow-ups could contribute significantly in reducing pregnancy complications and adverse pregnancy outcomes [5].

Previous studies identified serum *β*-human chorionic gonadotropin (*β*-hCG) as a key parameter associated with the incidence of pregnancy complications and adverse pregnancy outcomes [6–8]. Serum *β*-hCG is a glycoprotein secreted by placental trophoblast cells [7, 9, 10]. Embryo formation happens after fertilized egg moves into the uterine cavity and implants in matured woman. During the development of fetus, the placental syncytiotrophoblast produce a large amount of HCG which could be excreted into urine through maternal blood circulation. Serum and urine HCG levels rapidly increase from 1 to 2.5 weeks of gestation, peak at the 8th week of pregnancy, decrease to moderate levels by the 4th month of pregnancy, and remain at the end of pregnancy [11, 12]. Serum *β*-hCG levels can reflect the functional status of the placenta. When placental ischemia and hypoxia happen, the secretion of *β*-hCG by trophoblast cells increases [13–15]. Placental function is directly related to the occurrence of various diseases during pregnancy and adverse pregnancy outcomes.

However, the correlation of *β*-hCG and the incidence of pregnancy complications and adverse pregnancy outcomes have been controversial. Some studies suggested that high levels of *β*-hCG have no significant correlation with the incidence of IUGR, PIH, PD, and GDM [16]. In contrary, other studies concluded that high levels of *β*-hCG in the second trimester predict a high incidence of complications during pregnancy and poor pregnancy outcomes [17, 18]. Through our meta-analysis, we discovered that the inconsistent results might be caused by different study design, subject bias due to inconsistent demographic characteristics, and difference in medical levels. This study is aimed at elucidating the correlation of high levels of *β*-hCG on pregnancy complications and adverse pregnancy outcomes through a meta-analysis.

## 2. Materials and Methods

### 2.1. Literature Download

Literature searches were performed in PubMed, EMBASE, Medline, Central, China National Knowledge Infrastructure (CNKI), Wanfang, and China Science Digital Library (CSDL) databases. Searching terminology were as follows: *β*-human chorionic gonadotropin or *β*-hCG or hCG or human chorionic gonadotropin and pregnancy and adverse outcomes or complications. The languages of the literature are English and Chinese. The retrieval date was July 1, 2022.

### 2.2. Literature Screening

Inclusion criteria were as follows: (1) research subjects were singleton pregnant women; (2) the study is identified as cohort study; (3) the subjects were assigned to the high *β*-hCG group and control group according to whether the exposure factors increased *β*-hCG in the second trimester; (4) the observed outcomes include at least pregnancy-induced hypertension (PIH), diabetes (gestational diabetes mellitus, GMD), preterm delivery (PD), and intrauterine growth restriction (IUGR); and (5) the odds ratio (OR) and 95% confidence interval (CI) of exposure factors are calculated based on literature dataset.

Exclusion criteria were as follows: (1) repeated reports, (2) animal experiments, (3) inconsistent study types, (4) no control group, (5) inconsistent outcome indicators, and (6) incomplete literature data with authors contacted but data not replenished.

### 2.3. Data Extraction and Literature Risk Bias Assessment

Literature screening was performed by two researchers jointly. Data including author, title, publication time, study type, study number, number of high *β*-hCG group, number of control group, number of PIH, number of diabetes mellitus, number of intrauterine development, the number of delays, the number of miscarriages, and the number of premature births were extracted from included literatures. Unavailable datasets were obtained by contacting the authors. Two researchers performed the Newcastle-Ottawa Scale (NOS) to assess the risk of bias in the included studies, including the selectivity, comparability, and exposure factors and outcomes of study methods. NOS score ≥ 6 was classified as low risk of bias, otherwise, high risk of bias. During the process of data extraction and risk of literature bias assessment, if there was disagreement between the researchers, consensus was reached through discussion.

### 2.4. Statistical Methods

Cochrane software RevMan5.3 was used for statistical analysis in this study. The OR value was calculated by the number of cases and the number of cases in the group. Statistical descriptions of effect sizes were performed using OR values and 95% CIs. Heterogeneity was determined using chi-square test. When the degree of freedom corrected *I*^2^ > 50% or *P* < 0.1, it was considered that there was heterogeneity among the published literatures. Subgroup analysis was used to explore the root cause of heterogeneity. When heterogeneity could not be eliminated, use a random effects model or review only. When the degree of freedom corrected *I*^2^ ≤ 50% and *P* ≥ 0.1, it was considered that there was no heterogeneity among the publications, and a fixed effect model was used. Publication bias was assessed using funnel plots and Egger's test. Two-sided *P* < 0.05 indicates statistical significance.

## 3. Results

### 3.1. Basic Features of the Included Literature

**Figure 1:**
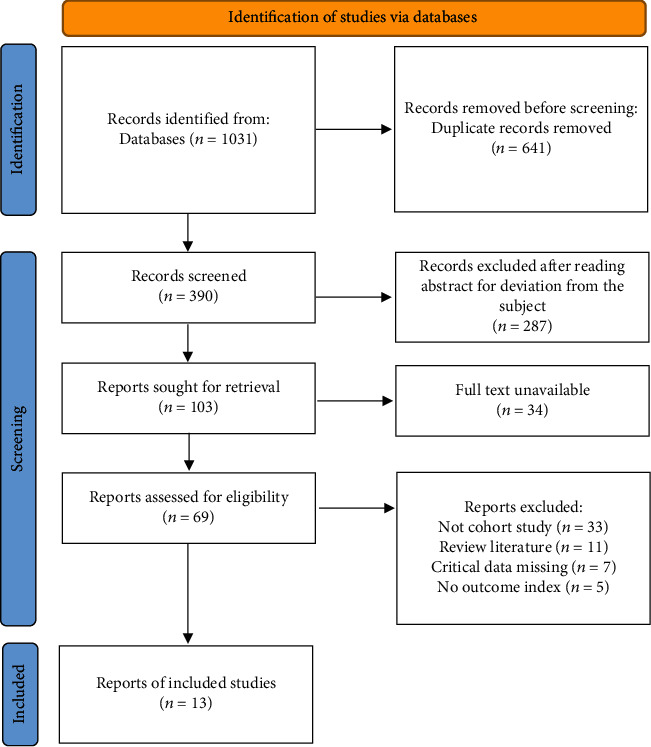
Flowchart of literature screening.

In this study, a total of 1031 literatures were retrieved from the above databases, 1018 literatures were excluded, and 13 literatures were included for this study [16–28]. The literature screening flowchart was shown in Figure 1. The 13 articles included 67355 pregnant women, among which, 5980 pregnant women were in the high *β*-hCG group and 61375 pregnant women were in the control group. All included literature information is shown in Table 1. The risk of bias assessment in the literature was shown in Table 2.

### 3.2. High *β*-hCG and PIH

**Figure 2:**
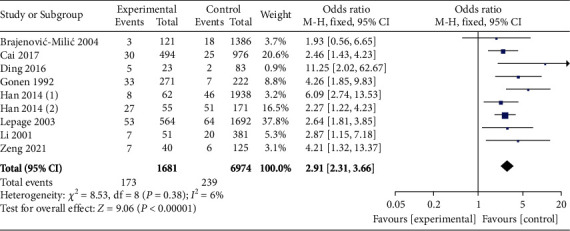
Forest plot: the incidence of pregnancy-induced hypertension in the high *β*-hCG group and the control group.

**Figure 3:**
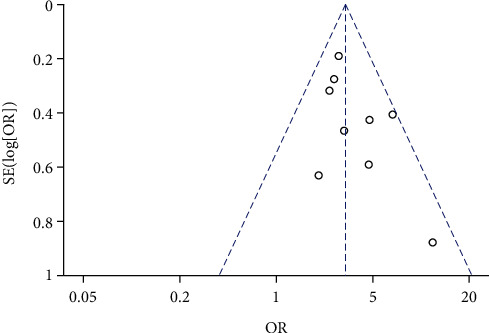
Funnel plot: the incidence of pregnancy-induced hypertension in the high *β*-hCG group compared with the control group.

A total of 9 studies compared the incidence of PIH between high *β*-hCG and control groups. No heterogeneity among 9 studies were identified from heterogeneity test (*χ*^2^ = 8.53, *P* = 0.38, *I*^2^ = 6%). Therefore, a fixed-effects model was used for pooling. As shown in Figure 2, the incidence of PIH was suggested to be significantly higher in the high *β*-hCG group than in the control group (OR = 2.91, 95% CI [2.31, 3.66], *Z* = 13.85, *P* < 0.00001). Egger's test and funnel plot shown in Figure 3 showed that the scatter points were distributed within the confidence interval with a semisymmetrical shape, and no publication bias was found (*P* > 0.05).

### 3.3. High *β*-hCG and PD

**Figure 4:**
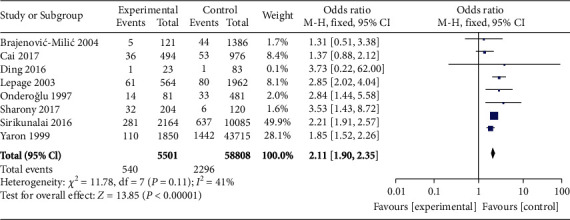
Forest plot: comparison of the incidence of premature delivery in the high *β*-hCG group and the control group.

**Figure 5:**
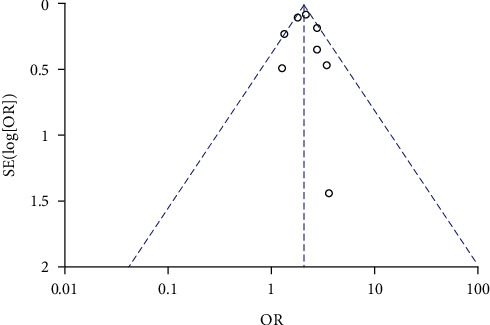
Funnel plot: comparison of the incidence of premature delivery in the high *β*-hCG group and the control group.

A total of 8 studies compared the incidence of PD between high *β*-hCG and control groups. The heterogeneity test confirmed nonheterogeneity among these studies (*χ*^2^ = 11.78, *P* = 0.11, *I*^2^ = 41%). Therefore, a fixed-effects model was used for pooling. Compared to the control group, the analyzed results showed higher incidence of PD in the high *β*-hCG group (OR = 2.11, 95% CI [1.90, 2.35], *Z* = 13.85, *P* < 0.00001), as shown in Figure 4. Semisymmetrical distribution of scatter points within the confidence interval was observed, as shown in Figure 5, using Egger's test and funnel plot.

### 3.4. High *β*-hCG and Abortion

**Figure 6:**
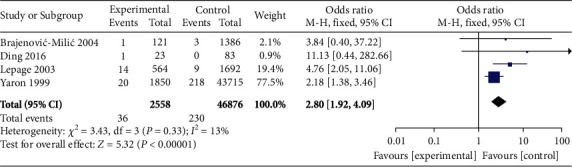
Forest plot: comparison of the incidence of miscarriage in the high *β*-hCG group and the control group.

**Figure 7:**
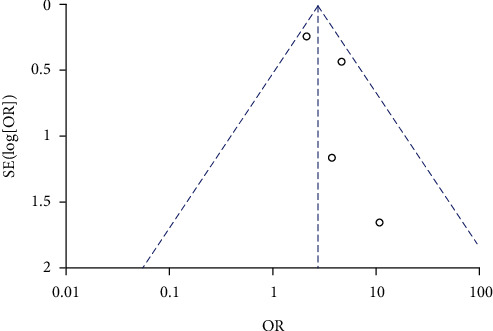
Funnel plot: comparison of the incidence of miscarriage between the high *β*-hCG group and the control group.

A total of 4 studies compared the incidence of miscarriage between high *β*-hCG and control groups. There was no heterogeneity among selected 4 studies basing on the heterogeneity test (*χ*^2^ = 3.43, *P* = 0.33, *I*^2^ = 13%). Therefore, a fixed-effects model was used for pooling. The analysis results showed that the incidence of miscarriage in the high *β*-hCG group was higher than that in the control group (OR = 2.80, 95% CI [1.92, 4.09], *Z* = 5.32, *P* < 0.00001), as shown in Figure 6. The scatter points fell in range of the confidence interval using Egger's test and funnel plot along with a semisymmetrical shape (*P* > 0.05) as shown in Figure 7.

### 3.5. High *β*-hCG and GDM

**Figure 8:**
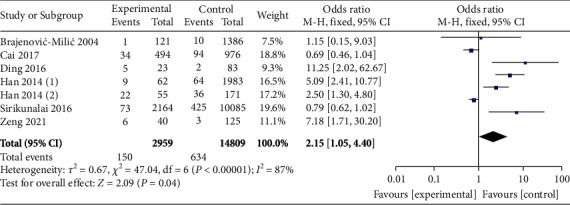
Forest plot: the incidence of gestational diabetes mellitus in the high *β*-hCG group compared with the control group.

**Figure 9:**
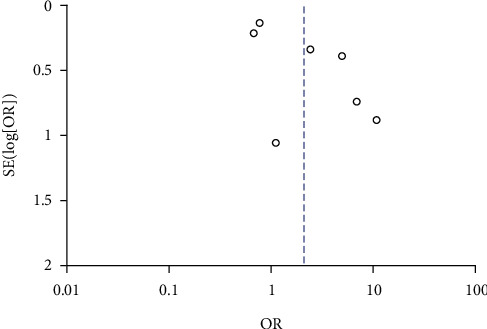
Funnel plot: incidence of gestational diabetes mellitus in the high *β*-hCG group compared to the control group.

A total of 7 studies compared the incidence of GDM between high *β*-hCG and control groups. A heterogeneity was identified among the 7 studies with heterogeneity test (*χ*^2^ = 47.01, *P* < 0.00001, *I*^2^ = 87%). Therefore, a random effects model was used for pooling. The analysis results showed that the incidence of GDM during pregnancy in the high *β*-hCG group was higher than that in the control group (OR = 2.15, 95% CI [1.05, 4.40], *Z* = 2.09, *P* = 0.04), as shown in Figure 8. Sensitivity analysis showed that the results were not robust, as shown in Table 3. Egger's test and funnel plot showed that the scatter points were distributed within the confidence interval, roughly symmetrical, and there was no publication bias (*P* > 0.05), as shown in Figure 9.

### 3.6. High *β*-hCG and IUGR

**Figure 10:**
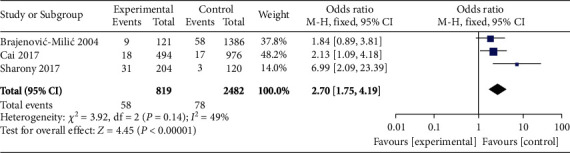
Forest plot: comparison of the incidence of intrauterine growth restriction in the high *β*-hCG group and the control group.

**Figure 11:**
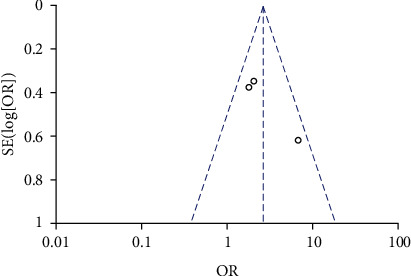
Funnel plot: the incidence of intrauterine growth restriction in the high *β*-hCG group compared with the control group.

A total of 3 studies compared the incidence of IUGR between high *β*-hCG and control groups. The included 3 studies did not exhibit heterogeneity (*χ*^2^ = 3.92, *P* = 0.14, *I*^2^ = 49%). Therefore, a fixed-effects model was used for pooling. The analysis results showed that the incidence of IUGR in the high *β*-hCG group was higher than that in the control group (OR = 2.70, 95% CI [1.75, 4.19], *Z* = 4.45, *P* < 0.0001), as shown in Figure 10. Egger's test and funnel plot showed that the scatter points were distributed semisymmetrically within the confidence interval, and there was no publication bias (*P* > 0.05), as shown in Figure 11.

## 4. Discussion

Through our comprehensive meta-analysis, we concluded that high levels of *β*-hCG are risk factors for IUGR, PIH, PD, and miscarriage in singleton pregnancy. In terms of GDM, sensitivity analysis showed that the results were not robust. The relationship between high levels of *β*-hCG and GDM still needs further research to confirm.

High levels of *β*-hCG in the second trimester predict a high incidence of complications during pregnancy and poor pregnancy outcomes [9]. The possible underlying mechanism is that *β*-hCG produced by placental trophoblasts can directly reflect placental function, and placental function is directly related to the occurrence of various diseases during pregnancy and adverse pregnancy outcomes [29]. At present, it is believed that GDM, PIH, IUGR, PD, miscarriage, fetal respiratory distress, and stillbirth are all caused by placental pathophysiological changes [29, 30]. Taken all together, the use of *β*-hCG to predict the occurrence of gestational hypertension has its pathophysiological basis.

Brajenović-Milić et al. [16] studied that elevated *β*-hCG levels could lead to an increased incidence of preeclampsia; however, elevated *β*-hCG levels were not identified as an independent risk factor for preeclampsia. There is no significant evidence which suggests that high levels of were associated with the incidence of IUGR, PIH, PD, and GDM. We analyzed that the study was biased in the selection of pregnant women. Pregnant women in the study and control groups were poorly balanced with respect to baseline data. Sirikunalai et al. [17] suggested that low or high *β*-hCG levels will increase the risk of complications and adverse outcomes for pregnant women. However, this conclusion only fitted in the second trimester. Lepage et al. [18] showed that in singleton pregnant women, high *β*-hCG levels were associated with a high incidence of pregnancy complications. In multiple pregnancies, the conclusion remains consistent. Sharony et al. [26] found a strong correlation between high levels of *β*-hCG and the incidence of IUGR and PD. However, the morbidities of several other complications, including preeclampsia, placental abruption, and prenatal death, were associated with extremely high levels of beta-hCG, suggesting that pregnant women with high levels of beta-hCG should be counseled and monitored in extreme case. Cai et al. [19] found that there was no significant difference in the incidence of gestational hypertension, fetal distress, and placental abruption between pregnant women aged ≥35 years and the elevated *β*-hCG group compared with the normal group. The increase of serum *β*-hCG level in the second trimester is closely related to the occurrence of adverse pregnancy outcomes, and it has certain clinical significance in predicting adverse pregnancy outcomes in obstetrics in combination with maternal age. Ding et al. [20] found that the incidence of gestational diabetes mellitus, gestational hypertension, oligohydramnios, and neonatal asphyxia in pregnant women with high serum *β*-hCG levels was significantly higher than those in women with normal levels. In addition, the same trend was not observed in the low *β*-hCG group and the normal group.

With the comprehensive meta-analysis, this study does exhibit some limitations. First of all, all included studies have inconsistent criteria for the definition of high *β*-hCG, which may affect the robustness of the results. Second, there is heterogeneity among studies in the relationship between *β*-hCG and GDM incidence; however, the root cause of heterogeneity was not identified. Thirdly, we did not explore the effect of very high or low levels of *β*-hCG on the incidence of pregnancy complications and adverse pregnancy outcomes. Ghasemi-Tehrani et al. [31] found that low levels of *β*-hCG has no significant effect on the incidence of complications including PD, PIH, miscarriage, and IUGR. Another study [32] pointed out that very high levels of *β*-hCG increase the risk of adverse outcomes in pregnant women, including stillbirth, small-for-gestational-age infants, and complete moles. Finally, we were not able to age-stratify pregnant women for more instructive results.

In conclusion, high levels of *β*-hCG during pregnancy in singleton women are associated with a high incidence of pregnancy complications and adverse pregnancy outcomes. Pregnant women with high levels of *β*-hCG should be monitored more closely, followed up, and given timely medical interventions to reduce the incidence of pregnancy complications and adverse outcomes.

## Figures and Tables

**Table 1:** Basic information of literature.

Author	Year	Study type	No. of patients		Outcomes	Standards of high HCG
			High *β*-hCG	Control		
Brajenović-Milić et al. [16]	2004	Cohort	121	1386	PIH, PD, abortion, GMD, IUGR	≥2.0 MoM
Cai et al. [19]	2017	Cohort	494	976	PIH, GMD, PD, IUGR	≥2.0 MoM
Ding et al. [20]	2016	Cohort	23	83	PIH	>2.0 MoM
Gonen et al. [21]	1992	Cohort	271	222	IUGR, PIH	>2.5 MoM
Han et al. [22]	2014	Cohort	62	1938	PIH, GMD	≥2.0 MoM
Han et al. [23]	2014	Cohort	55	171	PIH, GMD	>2.0 MoM
Lepage et al. [18]	2003	Cohort	564	1692	PD, PIH, abortion	≥2.0 MoM
Li et al. [24]	2001	Cohort	51	381	PIH	>2.0 MoM
Onderoğlu and Kabukcu [25]	1997	Cohort	81	481	PD	> 2 MoM
Sharony et al. [26]	2017	Cohort	204	120	IUGR, PD	> 3.0 MoM
Sirikunalai et al. [17]	2016	Cohort	2164	10085	PD, abortion	>2.0 MoM
Yaron et al. [27]	1999	Cohort	1850	43715	Abortion, PD	>2.5 MoM
Zeng et al. [28]	2021	Cohort	40	125	PIH, CMD	>2.0 MoM

Note: PIH: pregnancy-induced hypertension; GMD: gestational diabetes mellitus; PD: premature delivery; IUGR: intrauterine growth restriction; *β*-hCG: *β*-human chorionic gonadotropin; MoM: multiples of the median.

**Table 2:** Literature risk of bias assessment.

Study	Selection				Comparability control for important factor	Exposure			NOS
	Adequate definition of case	Representativeness of the case	Selection of controls	Definition of controls		Ascertainment of exposure	Same method of ascertain for cases and controls	Nonresponse rate	
Brajenović-Milić	^∗^	—	—	^∗^	—	^∗^	^∗^	—	4
Cai	^∗^	^∗^	^∗^	^∗^	^∗^	—	^∗^	^∗^	7
Ding	^∗^	—	^∗^	^∗^	—	^∗^	^∗^	^∗^	6
Gonen	^∗^	—	^∗^	^∗^	—	—	^∗^	^∗^	5
Han (1)	^∗^	^∗^	^∗^	^∗^	^∗^	—	^∗^	^∗^	7
Han (2)	^∗^	^—^	^∗^	^∗^	—	^∗^	^∗^	^∗^	7
Lepage	^∗^	^∗^	^∗^	^∗^	—	^∗^	^∗^	^∗^	7
Li	^∗^	—	^∗^	^∗^	^∗^	—	^∗^	—	5
Onderoğlu	^∗^	—	^∗^	^∗^	—	^∗^	^∗^	—	5
Sharony	^∗^	^∗^	^∗^	^∗^	—	—	^∗^	^∗^	6
Sirikunalai	^∗^	—	^∗^	^∗^	^∗^	^∗^	^∗^	^∗^	7
Yaron	^∗^	—	^∗^	^∗^	—	—	^∗^	^∗^	5
Zeng	^∗^	^∗^	^∗^	^∗^	^∗^	—	^∗^	^∗^	7

**Table 3:** Sensitivity analysis between high *β*-hCG and GDM.

Eliminate literature	Heterogeneity	OR	*P* value	*Z*
Brajenović-Milić et al. [16]	*I* ^2^ = 89%, *P* < 0.00001	2.28	0.03	2.13
Cai et al. [19]	*I* ^2^ = 88%, *P* < 0.00001	2.92	0.03	2.16
Ding et al. [20]	*I* ^2^ = 87%, *P* < 0.00001	1.79	0.11	1.61
Han et al. [22]	*I* ^2^ = 82%, *P* < 0.00001	1.69	0.12	1.54
Han et al. [23]	*I* ^2^ = 87%, *P* < 0.00001	2.10	0.07	1.81
Sirikunalai et al. [17]	*I* ^2^ = 86%, *P* < 0.00001	2.87	0.04	2.09
Zeng et al. [28]	*I* ^2^ = 87%, *P* < 0.00001	1.83	0.10	1.63

## Data Availability

The data used and analyzed during the current study are available from the corresponding author.
